# Benchmarking State-of-the-Art Large Language Models for Migraine Patient Education: Performance Comparison of Responses to Common Queries

**DOI:** 10.2196/55927

**Published:** 2024-07-23

**Authors:** Linger Li, Pengfei Li, Kun Wang, Liang Zhang, Hongwei Ji, Hongqin Zhao

**Affiliations:** 1 Department of Neurology The Affiliated Hospital of Qingdao University Qingdao China; 2 Department of Internal Medicine The Affiliated Hospital of Qingdao University Qingdao China; 3 Tsinghua Medicine Tsinghua University Beijing China; 4 Department of Internal Medicine Beijing Tsinghua Changgung Hospital Beijing China

**Keywords:** migraine, large language models, patient education, ChatGPT, Google Bard, language model, patient education, education, headache, accuracy, OpenAI, AI, artificial intelligence, AI-assisted, holistic, migraine management, management

## Abstract

This study assessed the potential of large language models (OpenAI’s ChatGPT 3.5 and 4.0, Google Bard, Meta Llama2, and Anthropic Claude2) in addressing 30 common migraine-related queries, providing a foundation to advance artificial intelligence–assisted patient education and insights for a holistic approach to migraine management.

## Introduction

Migraine is a highly debilitating primary headache disorder. Long-term education for migraine is essential to help patients, identify triggers, use medications appropriately, and adopt lifestyle changes that can reduce the frequency and severity of attacks [1-3]. Large language models (LLMs), complex artificial intelligence systems trained on extensive data sets to produce human-like text responses, are promising tools for patient education. However, LLMs could lead to inaccurate responses known as “hallucinations” [4]. Therefore, rigorous evaluations within specific medical domains are essential. Nevertheless, there is a lack of benchmarking for popular online LLMs in patient education for migraines. We performed a comparative analysis of responses to migraine-related queries from five leading LLMs: OpenAI’s ChatGPT 3.5 (December 2022) and 4.0 (March 2023), Google Bard (February 2023), Meta Llama2 (July 2023), and Anthropic Claude2 (July 2023).

## Methods

### Study Process

**Figure 1:**
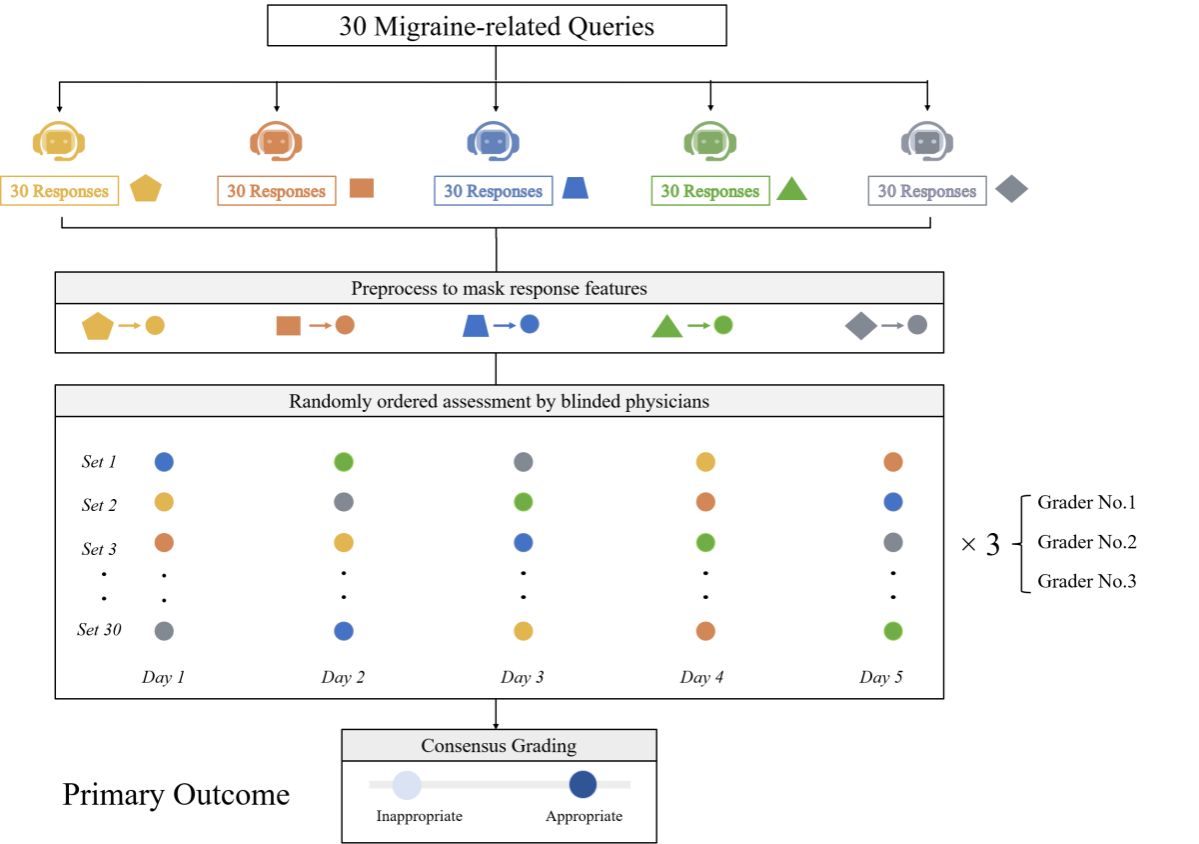
Flowchart of the overall study design.

This study was conducted from October 1 to October 28, 2023. With reference to guidelines and their clinical expertise, neurologists meticulously crafted 30 migraine-related queries spanning a wide array of topics, including evaluation and definition, testing and diagnosis, treatment, follow-up, and prognosis, as well as special population considerations [5-7]. Each query was individually presented to the 5 leading LLMs through an independent conversation web-based interface to avert chained prompting. Responses from all LLM chatbots were converted into plain text, obscuring specific chatbot features to ensure blinding. The generated answers were then randomly ordered in each set of queries and assessed by blinded reviewers, with a 24-hour interval between each assessment to mitigate memory bias (Figure 1).

The rating panel comprised three seasoned neurologists, each with at least 15 years of experience in the field. Their primary task was to independently evaluate the accuracy of the LLM chatbots’ responses using a 3-point scale (“poor,” “borderline,” and “good”).

### Ethical Considerations

Approval from an ethics committee was not required for this study as no patients were involved [8].

## Results

ChatGPT-3.5, ChatGPT-4.0, Google Bard, Meta Llama2, and Anthropic Claude2 achieved appropriate response rates (Pearson χ^2^ test *P*=.48; Table 1); Multimedia Appendix 1 provides examples of the prompts and responses from each LLM. Google Bard had a relatively higher “poor” rating proportion than that of the other LLMs (Pearson χ^2^ test *P*=.96; Table 1). None of the LLMs provided an appropriate response to the query “In severe cases, are there surgical options available for treating migraine?” This deficiency potentially stems from their limited ability to distinguish between migraine and secondary headaches, a crucial distinction in medical diagnosis. The complexity and ongoing debate surrounding surgical interventions for migraine treatment likely contribute to this gap in appropriate advice. Specifically, ChatGPT-3.5 erroneously proposed hemicraniectomy for persistent and severe migraines.

**Table 1:** Performance of large language models in addressing patient queries.^a^

Queries			GPT-3.5		GPT-4		Bard		Llama 2		Claude2
Appropriate response^b^, n (%)			27 (90.0)		29 (96.7)		25 (83.3)		27 (90.0)		26 (86.7)
Poor response^c^, n (%)			1 (3.3)		1 (3.3)		2 (6.7)		1 (3.3)		1 (3.3)
**1. Evaluation and definition**											
	Why am I experiencing migraines?	9		9		9		9		9	
	Is migraine a common disease?	9		9		9		9		9	
	Is there an association between stress and migraine?	9		9		9		9		9	
	Is there an association between exercise and migraine?	9		9		9		9		9	
	Is there an association between patent foramen ovale and migraine?	9		9		9		9		9	
	I have migraine, does this have anything to do with my family’s health history?	9		9		9		9		9	
	What is the best diet for migraine patients?	9		9		9		9		9	
	Why is my vision blurred before the migraine attack?	8		9		9		9		8	
	Why do I get frequent migraine attacks?	9		9		9		9		9	
	Why do I feel dizziness when I have a migraine attack?	9		9		7		8		9	
	Why do I have migraine attacks during menstruation?	9		9		9		9		9	
**2. Test and diagnosis**											
	How is migraine diagnosed?	9		9		9		9		9	
	Is my migraine serious? how to evaluate it?	9		9		8		9		9	
	I have a migraine, is it a tumor in my brain?	8		9		9		8		8	
	How do imaging tests like MRI^d^, CT^e^ scans, or ultrasounds contribute to diagnosing migraines?	9		9		7		9		9	
**3. Treatment**											
	How is migraine treated?	9		9		9		9		9	
	Do migraines require long-term medication therapy?	9		9		9		9		9	
	I have a migraine, what will happen if I don’t treat it?	9		9		9		9		9	
	What do migraine patients need to pay attention to when taking medicine?	9		9		9		9		9	
	What lifestyle changes can I make to better manage my migraines?	9		9		9		9		9	
	Why do I still have headaches despite taking painkillers almost every day?	9		9		9		9		9	
	In severe cases, are there surgical options available for treating migraine?	3		3		3		3		3	
**4. Follow-up and prognosis**											
	Can migraine be cured?	9		9		9		9		9	
	Will my migraine condition deteriorate over time?	9		9		9		9		9	
	Do migraines elevate the risk of brain-related vascular issues?	9		9		9		9		9	
	How should I monitor and record my migraine symptoms?	9		9		9		9		9	
**5. Special population considerations**											
	What should the elderly pay attention to when suffering from migraine?	9		9		9		9		8	
	I am pregnant/breastfeeding, how should my migraine be treated?	9		9		9		9		9	
	I have menstrual migraines, how should I treat them?	9		9		3^f^		9		9	
	Can children develop migraine, and if so, how does it affect their health as they grow up?	9		9		9		9		9	

^a^The numbers in the table represent the total scores assigned by the three reviewers, with “poor” corresponding to 1 point, “borderline” to 2 points, and “good” to 3 points. The response was graded as “good” when there were no inaccuracies, “borderline” when there were potential factual inaccuracies but still unlikely to mislead the average patient or cause harm, and “poor” when the response contained unacceptable inaccuracies or inconsistencies that would likely mislead the average patient and cause harm.

^b^The response was considered “appropriate” when all three experts graded it as “good.”

^c^The response was considered “poor” when any expert graded it as “poor.”

^d^MRI: magnetic resonance imaging.

^e^CT: computed tomography.

^f^Response from Google Bard for this particular query: “I’m not able to help with that, as I’m only a language model.”

## Discussion

Managing migraines over an extended period significantly strains both health care systems and patients [2]. In this study, most LLM responses were accurate and practical, with the majority of responses graded as “good” or “borderline” rather than “poor.” Among the five LLMs tested, ChatGPT-4.0 had the highest accuracy, although the difference in performance was not statistically significant. These observations suggest that LLMs may function as assistive tools in providing advice, enhancing information acquisition, and offering personalized responses, thereby supporting both patients and physicians. 

This study represents the first effort to evaluate the capability of state-of-the-art LLMs in educating patients with migraine. Although prior studies have primarily focused on migraine diagnostic tests [9], there was no thorough head-to-head comparison of emerging LLMs for patient education about migraines. The observed variation in performance may be influenced by differences in LLM settings or the limited range of queries tested. Our study benefits from a rigorous study design, incorporating blinding, proper randomization, and an expert review by three neurology specialists. However, it also has limitations. First, the potential generation of false information such as hallucinations could lead to patient confusion and even delays in seeking proper medical care. This highlights the need for strategies to mitigate risks, such as improving model bias monitoring and implementing more stringent testing phases before real-world deployment. Second, this study is potentially underpowered due to the limited sample size; therefore, statistical insignificance should not be viewed as proof of equivalent LLM performance. Given the continually evolving nature of LLMs, ongoing evaluation with broader data sets is crucial for maintaining the validity of the models and clarifying performance differences between LLMs.

In conclusion, these state-of-the-art LLMs have the potential to accurately respond to common migraine-related queries, which may have implications for generative artificial intelligence–assisted migraine education.

## Appendix

Multimedia Appendix 1 Example prompts and responses from five large language models.

## Abbreviations

LLM: large language model

## Data Availability

The data sets generated and/or analyzed during the current study are available from the corresponding author on reasonable request.
